# PXDN reduces autophagic flux in insulin-resistant cardiomyocytes via modulating FoxO1

**DOI:** 10.1038/s41419-021-03699-4

**Published:** 2021-04-26

**Authors:** Chan Li, Zhaoya Liu, Qian Xu, Huihui Peng, Jing Cao, Honghua Zhou, Guogang Zhang, Guangjie Cheng, Ruizheng Shi

**Affiliations:** 1 Department of Cardiovascular Medicine, Xiangya Hospital, Central South University, Changsha, Hunan China; 2 Department of the Geriatrics, The Third Xiangya Hospital, Central South University, Changsha, Hunan China; 3 Department of Cardiovascular Surgery, Xiangya Hospital, Central South University, Changsha, Hunan China; 4 Department of Cardiovascular Medicine, The Affiliated Puren Hospital of Wuhan University of Science and Technology, Wuhan, Hubei China; 5 Department of Cardiovascular Medicine, The Third Xiangya Hospital, Central South University, Changsha, Hunan China; 6 Department of Cardiovascular Medicine, Xiangya Hospital, Central South University, Changsha, Hunan China; 7 Department of Cardiovascular Medicine, The Third Xiangya Hospital, Central South University, Changsha, Hunan China; 8 Division of Pulmonary, Allergy and Critical Care Medicine, Department of Medicine, University of Alabama at Birmingham, Birmingham, AL USA; 9 Department of Cardiovascular Medicine, Xiangya Hospital, Central South University, Changsha, Hunan China

**Keywords:** Macroautophagy, RNAi

## Abstract

Autophagy, a well-observed intracellular lysosomal degradation process, is particularly important to the cell viability in diabetic cardiomyopathy (DCM). Peroxidasin (PXDN) is a heme-containing peroxidase that augments oxidative stress and plays an essential role in cardiovascular diseases, while whether PXDN contributes to the pathogenesis of DCM remains unknown. Here we reported the suppression of cell viability and autophagic flux, as shown by autophagosomes accumulation and increased expression level of LC3-II and p62 in cultured H9C2 and human AC16 cells that treated with 400 μM palmitate acid (PA) for 24 h. Simultaneously, PXDN protein level increased. Moreover, cell death, autophagosomes accumulation as well as increased p62 expression were suppressed by PXDN silence. In addition, knockdown of PXDN reversed PA-induced downregulated forkhead box-1 (FoxO1) and reduced FoxO1 phosphorylation, whereas did not affect AKT phosphorylation. Not consistent with the effects of si-PXDN, double-silence of PXDN and FoxO1 significantly increased cell death, suppressed autophagic flux and declined the level of FoxO1 and PXDN, while the expression of LC3-II was unchanged under PA stimulation. Furthermore, inhibition of FoxO1 in PA-untreated cells induced cell death, inhibited autophagic flux, and inhibited FoxO1 and PXDN expression. Thus, we come to conclusion that PXDN plays a key role in PA-induced cell death by impairing autophagic flux through inhibiting FoxO1, and FoxO1 may also affect the expression of PXDN. These findings may develop better understanding of potential mechanisms regarding autophagy in insulin-resistant cardiomyocytes.

## Introduction

Diabetic cardiomyopathy (DCM), promoted by insulin resistance, hyperlipidema, and hyperglycaemia^1^, is described as the existence of abnormal myocardial structure and function independent of recognized cardiac risk factors in individuals with diabetes^2^. As the progression of DCM, diastolic dysfunction often develops into systolic dysfunction, heart failure, and eventually cardiac death^3^, which accounts for significant morbidity and mortality in developed countries^4^. Multiple mechanisms are involved in the pathogenesis of DCM, among which recent researches have highlighted the importance of abnormal autophagy and subsequent cardiomyocyte death^5^.

Autophagy refers to a cellular protective mechanism that removes damaged proteins and organelles via lysosomal degradation pathways^6^. Impaired autophagy is associated with cardiomyocyte death and plays a key role in DCM^7,8^. In OVE26 diabetes mice, decreased AMPK activity and subsequent reduction of autophagy were correlated with cardiomyocyte death and cardiac dysfunction^9^. Drugs such as resveratrol, trehalose, curcumin, and liraglutide are demonstrated to reduce cell death by promoting autophagy in insulin-resistant cardiomyocytes and diabetic animals via various pathways^10–13^, Among which forkhead box-1 (FoxO1), a transcriptional factor, has been recognized as a key regulator. It can promote autophagy by stimulating autophagy-related genes and accelerate lysosomal proteolysis via rab7, MURF1, etc.^14,15^.

Peroxidasin (PXDN), also named vascular peroxidase 1 (VPO1), is a member of the heme-containing peroxidase family which is highly expressed in cardiovascular tissue and plays an important role in cardiovascular diseases^16–18^. Liu et al.^19^ found that PXDN was significantly increased in the aorta of type 2 diabetes rats. Previous studies also revealed that PXDN is capable to induce cell death via promoting apoptosis^20^ and programmed necrosis^21^ in endothelial cells. However, whether PXDN is involved in cardiomyocyte death in the diabetic heart has yet not been investigated.

In this study, we firstly investigated the role of PXDN in insulin-resistant H9C2 and AC16 cells, and our results suggest that PXDN inhibition may improve autophagic flux to reduce cell death in insulin-resistant cardiomyocytes. Moreover, PXDN was found to suppress the expression and dephosphorylation of FoxO1 under PA treatment while downregulaton of FoxO1 in turn inhibited PXDN expression. These findings may develop better understanding of potential mechanisms regarding autophagy in insulin-resistant cardiomyocytes.

## Results

### PXDN expression increases in the progress of cell death induced by PA

**Fig. 1:**
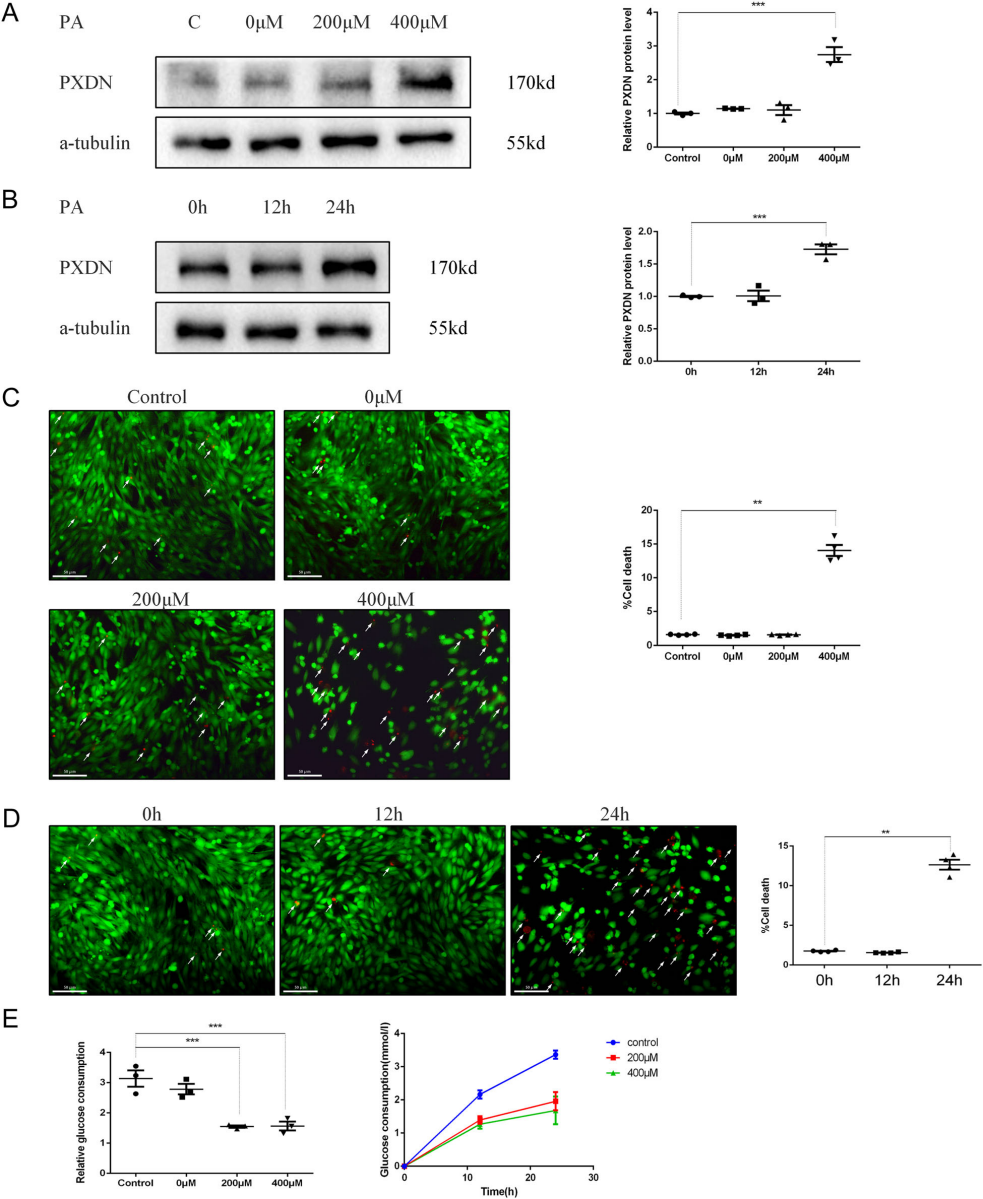
palmitate acid (PA) stimulates the expression of peroxidasin (PXDN) and promotes cell death in a time and dose manner. **A** H9C2 cells were treated with different concentrations of PA (0, 200, and 400 μM) for 24 h. PXDN protein level was measured by western blot and quantitative analysis was shown on the right side (*n* = 3). **B** H9C2 cells were treated with 400 μM PA for different duration (0, 12, and 24 h). PXDN protein level was measured and quantitative analysis was shown on the right side (*n* = 3). **C** H9C2 cells were treated with different concentrations of PA for 24 h; cell death rate was measured by LIVE/DEAD viability kit. Live cells were stained by vital dyes calcein acetoxymethyl ester (Calcein-AM) and exhibited green fluorescence, while dead cells were stained by ethidium homodimer-1 (EthD-1) and exhibited red fluorescence. The percentage of cell death was shown on the right side (scale bar = 50 μM, *n* = 4). **D** Cells were treated with 400 μM PA for different duration; the cell death rate was measured by LIVE/DEAD viability kit. The percentage of cell death was shown on the right side (scale bar = 50 μM, *n* = 4). **E** Cells were treated with different concentrations of PA for different duration, and glucose consumption was determined (*n* = 3). Data were presented as mean ± SEM. One-way ANOVA test was used. *P < 0.05, **P < 0.01, ***P < 0.001.

To explore whether the expression of PXDN was affected in the progress of fatty acid-induced cell death, H9C2 was treated with 0, 200 μM, or 400 μM of PA for 0, 12, and 24 h, respectively. We found that PXDN increased significantly after treated with PA (400 μM) for 24 h (Fig. 1A and B). Increased cell death was also observed via cell viability analyses after treatment with PA (400 μM) for 24 h (Fig. 1C and D) in H9C2. Thus, 400 μM palmitate for 24 h was used for subsequent experiments. In addition, insulin resistance was induced in cultured H9C2 cells, as shown by a less glucose consumption at 12 and 24 h after 200 or 400 μM PA treatment compared with the control group (Fig. 1E).

### PXDN silence improves impaired autophagic flux and reduces subsequent cell death induced by PA

**Fig. 2:**
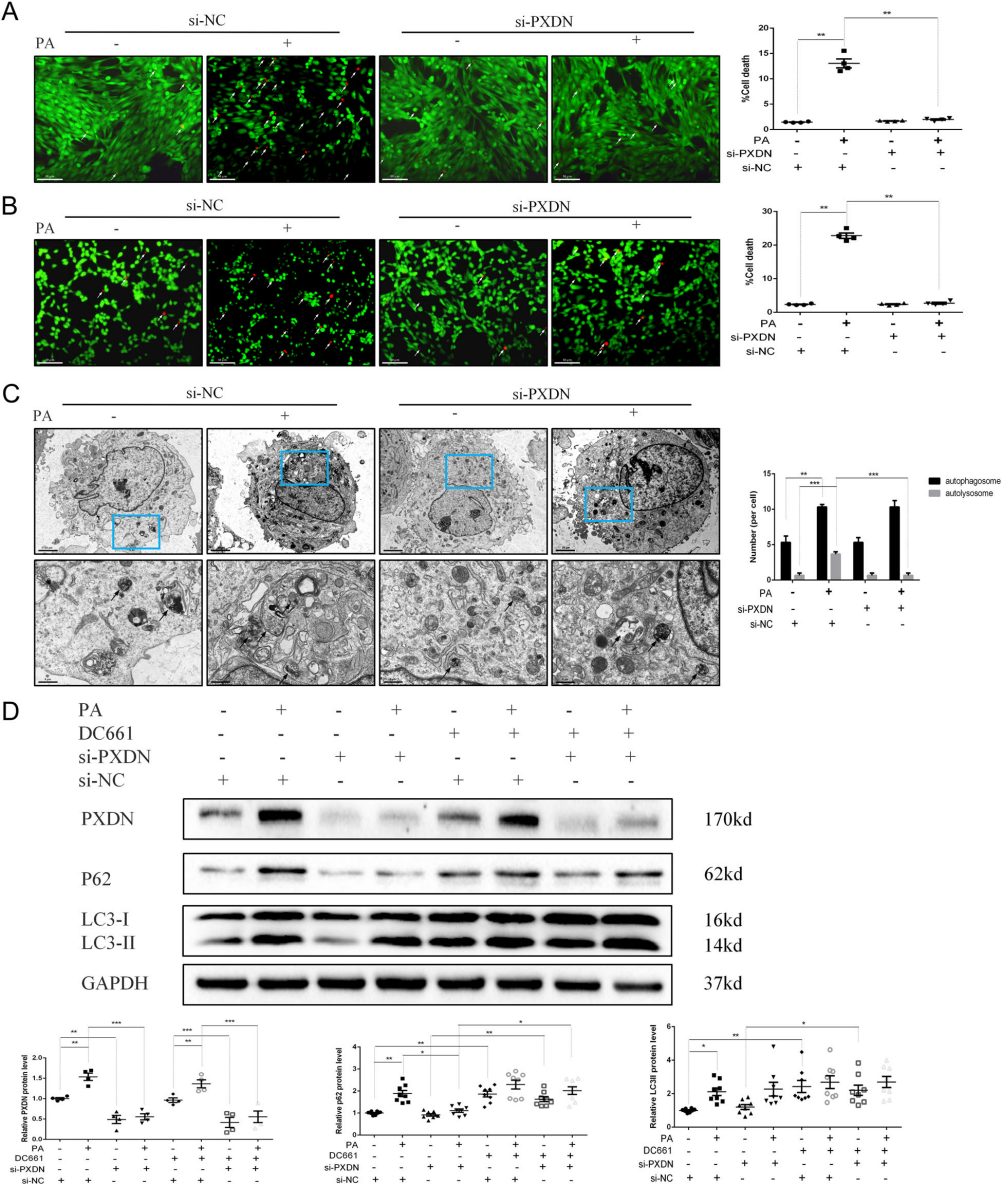
PXDN silence reduces cell death by promoting autophagy. **A** H9C2 cells were treated with 50 μM si-negative control (NC) or si-PXDN in serum-free medium for 24 h, and treated with 400 μM palmitate acid (PA) or bovine serum albumin (BSA) for another 24 h. Cell survival was evaluated using fluorescence staining with calcein acetoxymethyl ester (Calcein-AM) for live cells in green and ethidium homodimer-1 (EthD-1) for dead cells in red. The percentage of cell death was shown in the right side (scale bar = 50 μM, *n* = 4). **B** AC16 cells were treated with si-NC or si-PXDN in serum-free medium for 24 h, and treated with PA or BSA for another 24 h. Cell survival was evaluated using fluorescence staining. The percentage of cell death was shown in the right side (scale bar = 50 μM, *n* = 4). **C** Autophagosomes and autolysosomes in H9C2 with si-NC or si-PXDN in the absence or presence of PA were detected by transmission electron microscopy. Black Arrow referred to autophagosomes and white arrow referred to autolysosomes. The number of autophagosomes and autolysosomes per cell was shown in the right side (scale bar = 20 μm in top and 6 μm in bottom, respectively. *n* = 3). **D** H9C2 cells were treated with 50 μM si-NC or si-PXDN in serum-free medium for 24 h, and treated with 400 μM PA or BSA with or without 1 μM DC661 for another 24 h. PXDN, LC3II, and p62 levels were detected by Western blot and quantitative analyses were shown below (*n* = 4–8). Data were presented as mean ± SEM. One-way ANOVA test was used. *P < 0.05, **P < 0.01, ***P < 0.001.

Next, we explored whether PXDN was necessary for cell death stimulated by PA. siRNA was used to achieve PXDN inhibition in H9C2 cells before treated with PA. It was shown in Fig. 2D that PXDN is significantly knockdown by si-PXDN in H9C2 cells whether in control or PA-treated groups. Meanwhile, si-PXDN significantly reduced cell death under PA treatment (Fig. 2A). Transmission electron microscopy (TEM) was used to investigate whether autophagic flux was involved in PXDN-mediated cell death subsequently. A remarkable increase of both autophagosomes and autolysosomes was observed in PA group, while si-PXDN significantly decreased the number of autophagosomes (Fig. 2C). To further confirm whether PXDN impaired autophagosome turnover, DC661 was used to block autophagosome turnover by inhibiting lysosomal acidification. Immunoblotting showed that PA significantly increased the protein expression level of LC3II and p62. si-PXDN didn’t change LC3II and p62 levels in normal conditions, but significantly decreased the accumulation of p62 without affecting LC3II expression after PA. Treatment with DC661 increased the protein expression level of LC3II and p62 in the vehicle and si-PXDN group, but failed to further enhance LC3II and p62 expression level in the PA group. In addition, DC661 reversed the effect of si-PXDN on p62 after PA (Fig. 2D). Similar to H9C2 cells, the silence of PXDN significantly reduced cell death and decreased the accumulation of p62 under PA treatment in AC16 human cells (Fig. 2B and Fig. S1A).

These results revealed that PA treatment impaired autophagosome turnover and contributed to cell death in cardiomyocytes, while si-PXDN promoted autophagic flux and reduced cell death.

### PXDN silence enhances the expression and dephosphorylation of FoxO1

**Fig. 3:**
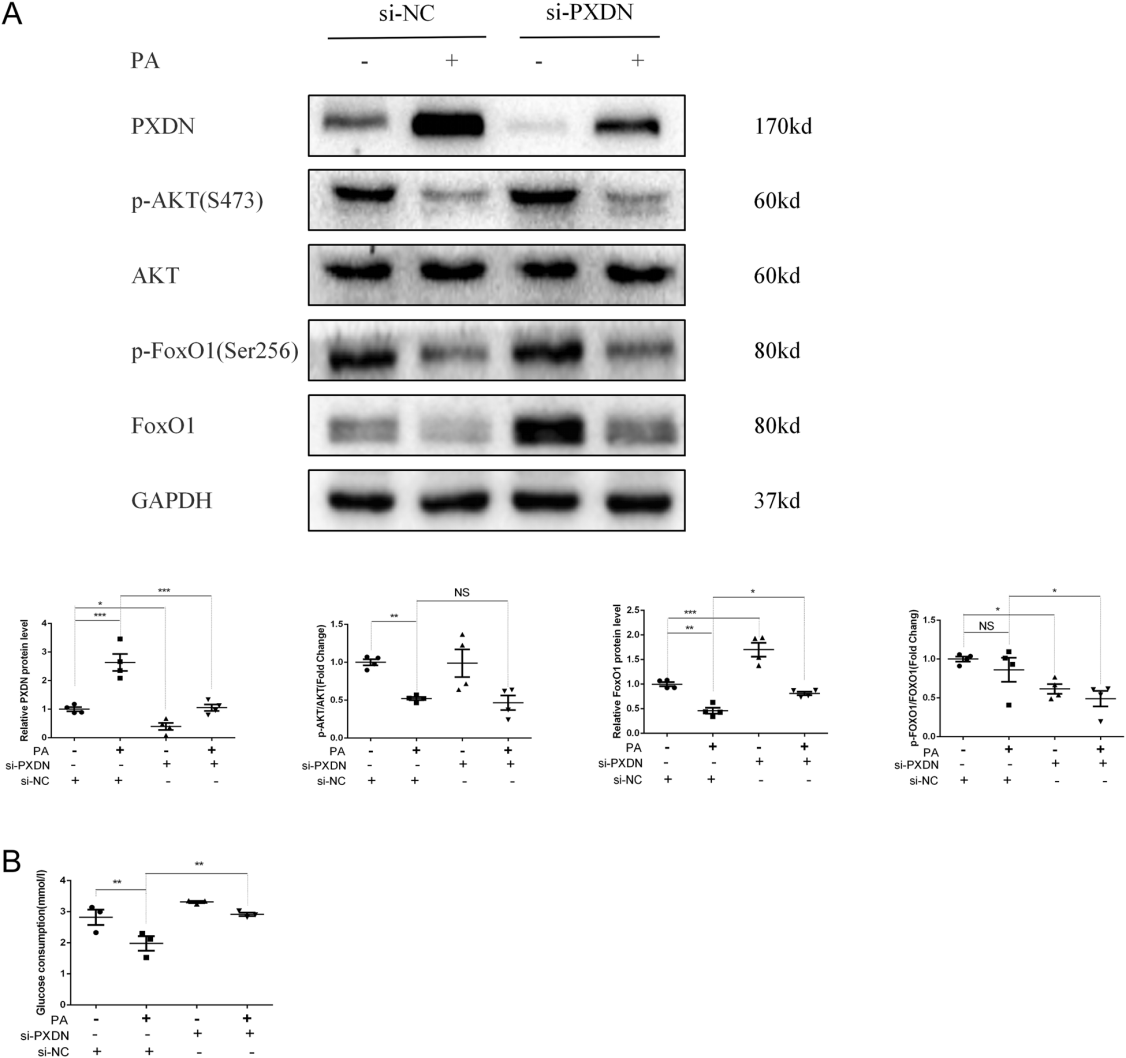
PXDN silence promotes the expression and activity of forkhead box-1 (FoxO1). **A** H9C2 cells were treated with 50 μM si-negative control (NC) or si-PXDN in serum-free medium for 24 h, and treated with 400 μM palmitate acid (PA) or bovine serum albumin (BSA) for another 24 h. p-AKT, AKT, p-FoxO1 and FoxO1 levels were detected by western blot and quantitative analyses were shown below (*n* = 4). **B** H9C2 cells were treated with si-NC or si-PXDN in the absence or presence of PA; glucose consumption was determined (*n* = 3). Data were presented as mean ± SEM. One-way ANOVA test was used. NS referred to no significance. *P < 0.05, **P < 0.01, ***P < 0.001.

To explore the potential pathways in PXDN regulating autophagic flux, we investigated several recognized regulators of autophagic flux including p-AKT/AKT and FoxO1 pathways. As shown in Fig. 3A, the decline of p-AKT/AKT after PA was not affected by si-PXDN treatment. However, si-PXDN restored the PA-induced decrease of FoxO1 protein level and reduced FoxO1 phosphorylation, indicating FoxO1 as a potential mediator between PXDN and autophagic flux. In addition, si-PXDN alleviated PA-induced insulin resistance in the form of increased glucose consumption (Fig. 3B).

### PXDN reduces autophagic flux to induce cell death via FoxO1 inhibition

**Fig. 4:**
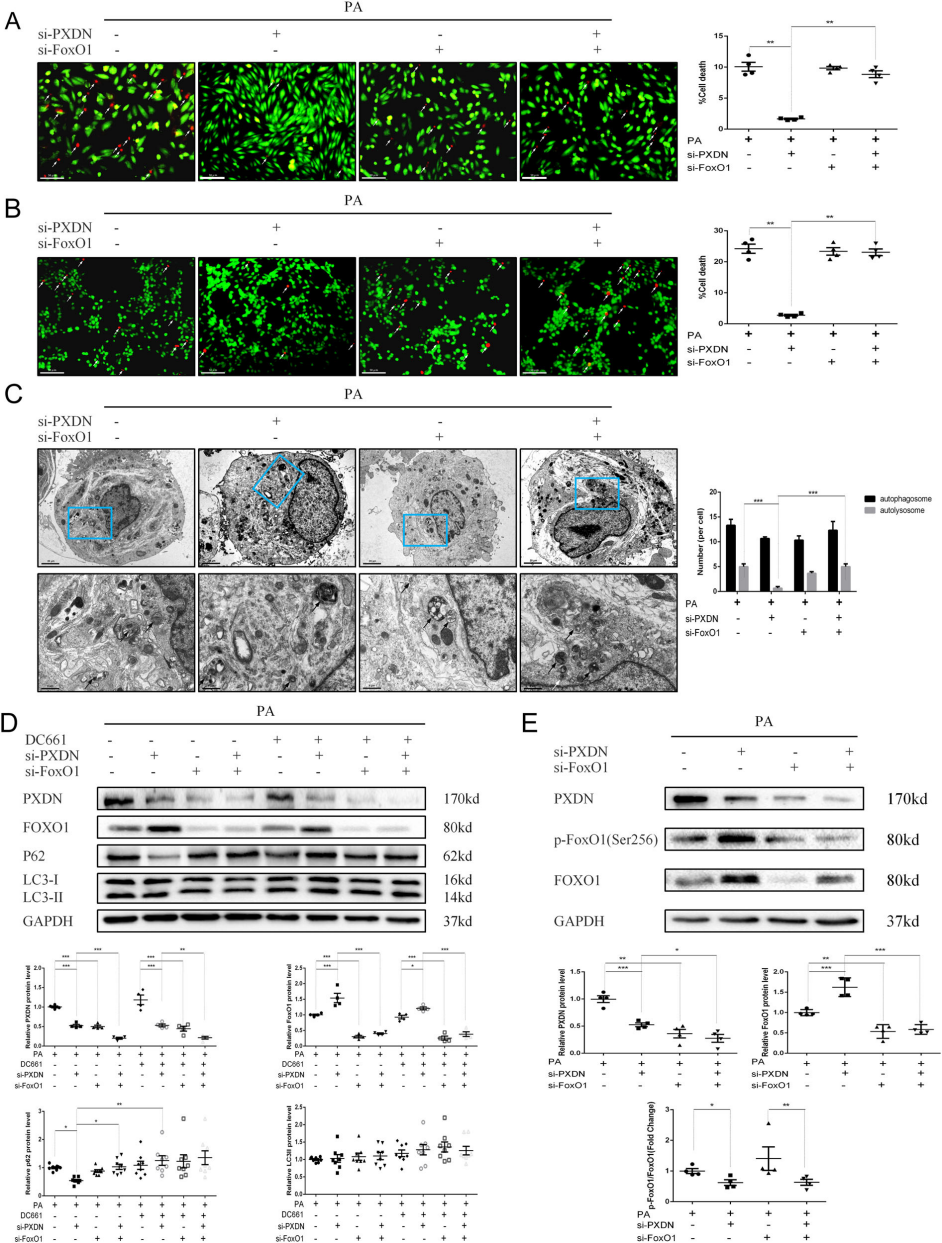
PXDN reduces autophagic flux via FoxO1 inhibition. **A** H9C2 cells were treated with 50 μM si-negative control (NC), si-PXDN, si-FoxO1, or si-PXDN plus si-FoxO1 in serum-free medium for 24 h, and treated with 400 μM palmitate acid (PA) for another 24 h. Cell survival was evaluated using fluorescence staining with calcein acetoxymethyl ester (Calcein-AM) for live cells in green and ethidium homodimer-1 (EthD-1) for dead cells in red. The percentage of cell death was shown on the right side (scale bar = 50 μM, *n* = 4). **B** AC16 cells were treated with NC, si-PXDN, si-FoxO1, or si-PXDN plus si-FoxO1 in serum-free medium for 24 h, and treated with 400 μM PA for another 24 h. Cell survival was evaluated using fluorescence staining. The percentage of cell death was shown on the right side (scale bar = 50 μM, *n* = 4). **C** Autophagosomes and autolysosomes in PA-treated H9C2 with si-NC, si-PXDN, si-FoxO1, or si-PXDN plus si-FoxO1 were detected by transmission electron microscopy. Black Arrow referred to autophagosomes and white arrow referred to autolysosomes. The number of autophagosomes and autolysosomes per cell was shown on the right side (scale bar = 20 μm in top and 6μm in bottom, respectively. *n* = 3). **D** H9C2 cells were treated with 50 μM si-NC, si-PXDN, si-FoxO1, or si-PXDN plus si-FoxO1 in serum-free medium for 24 h, and treated with 400 μM PA with or without 1 μM DC661 for another 24 h. PXDN, FoxO1, LC3II, and p62 levels were detected by western blot. Quantitative analyses were shown below (*n* = 4–8). **E** Western blot of PXDN、p-FoxO1, and FoxO1 in PA-treated H9C2 with si-NC, si-PXDN, si-FoxO1, or si-PXDN plus si-FoxO1 (*n* = 4). Quantitative analyses were shown below (*n* = 4). Data were presented as mean ± SEM. One-way ANOVA test was used. *P < 0.05, **P < 0.01, ***P < 0.001.

We further investigated the relationship between PXDN and FoxO1 in regulating autophagic flux and cell death in PA-treated cardiomyocytes. As shown in Figs. 4A and B, si-PXDN reduced cell death under PA treatment, while double silence of PXDN and FoxO1 blocked this effect in H9C2 and AC16 cells. Autophagic flux was measured subsequently. It was shown that, compared with downregulation of PXDN alone, double silence of PXDN and FoxO1 showed a severe autophagosome aggregation, while autolysosomes remained unchanged (Fig. 4C). LC3II protein level was basically unchanged between different groups under the stimulation of PA. si-PXDN significantly decreased the accumulation of p62, and this effect can be blocked by additional silence of FoxO1. DC661 didn’t further enhance p62 level except for si-PXDN group (Fig. 4D and Fig. S1B). These results suggested that FoxO1 may act as a downstream factor of PXDN in PA-medicated autophagosome accumulation and cell death.

As shown in Fig. 4E, additional FoxO1 silence could counteract the increase of FoxO1 induced by si-PXDN, while did not influence the phosphorylation of FoxO1. In contrast, FoxO1 phosphorylation decreased in the presence of si-PXDN. Interestingly, PXDN protein level decreased after treated with either si-PXDN or si-FoxO1, and was further downregulated while double silence. These results indicated that PXDN may suppress the expression and activity of FoxO1 under PA treatment while the silence of FoxO1 may also inhibit PXDN expression.

### FoxO1 inhibition induces cell death, reduces autophagic flux, and suppresses PXDN expression

**Fig. 5:**
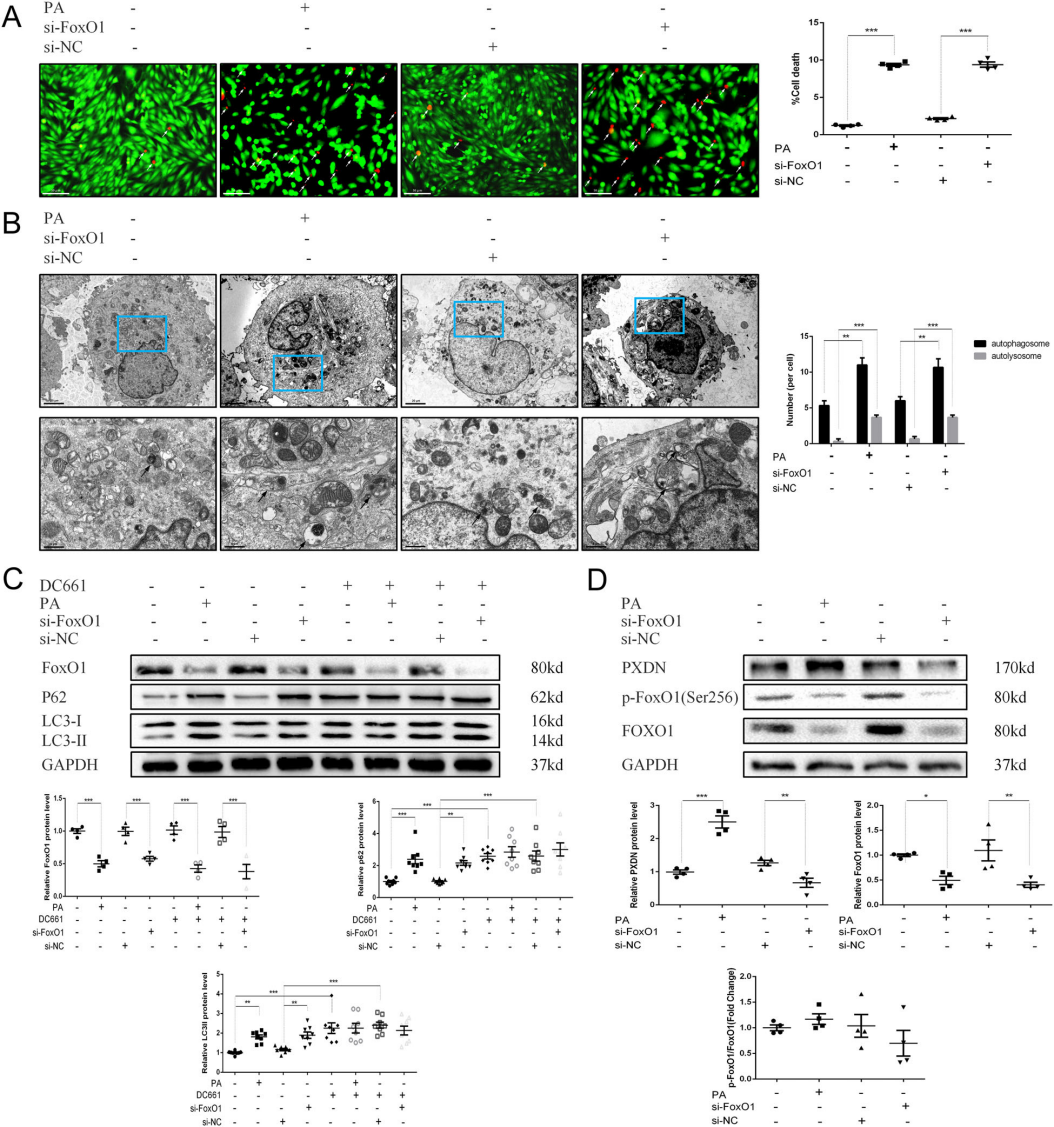
The effect of FoxO1 on PXDN. **A** H9C2 cells were treated with 50 μM si-negative control (NC), 50 μM si-FoxO1, 400 μM palmitate acid (PA), or 400 μM bovine serum albumin (BSA) for 24 h. Cell survival was evaluated using fluorescence staining with calcein acetoxymethyl ester (Calcein-AM) for live cells in green and ethidium homodimer-1 (EthD-1) for dead cells in red. The percentage of cell death was shown on the right side (scale bar = 50 μM, *n* = 4). **B** Autophagosomes and autolysosomes in PA-treated H9C2 with 50 μM NC, 50 μM si-FoxO1, 400 μM PA, or 400 μM BSA were detected by transmission electron microscopy. Black Arrow referred to autophagosomes and white arrow referred to autolysosomes. The number of autophagosomes and autolysosomes per cell was shown on the right side (scale bar = 20 μm in top and 6μm in bottom, respectively. *n* = 3). **C** H9C2 cells were treated with 50 μM si-NC, 50 μM si-FoxO1, 400 μM PA, or 400 μM BSA for 24 h (with or without 1 μM DC661). FoxO1, LC3II, and p62 levels were detected by western blot and quantitative analyses were shown below (*n* = 4–8). **D** H9C2 cells were treated with 50 μM NC, 50 μM si-FoxO1, 400 μM PA, or 400 μM BSA for 24 h. PXDN, p-FoxO1, and FoxO1 levels were detected by western blot and quantitative analyses were shown below (*n* = 4–8). Data were presented as mean ± SEM. One-way ANOVA test was used. *P < 0.05, **P < 0.01, ***P < 0.001.

To determine the effect of FoxO1 on cell death, autophagic flux, and PXDN under normal conditions, si-FoxO1 was used. It was shown in Fig. 5A–C that si-FoxO1, similar to PA stimulation, induced cell death and autophagosome accumulation, as well as up-regulated LC3II, p62 protein level, and downregulated FoxO1. However, unlike PA-induced PXDN up-regulation, si-FoxO1 significantly decreased the expression of PXDN. Co-immunoprecipitation (IP) was conducted subsequently and PXDN was not found in the IP products of FoxO1 antibody in both basal and PA conditions (Fig. S2). According to the JASPAR database, FoxO1 was predicted to bind with the promoter of PXDN gene (Table. S2). These results indicated that FoxO1 was indeed a key factor in autophagic flux regulation and could regulate the expression of PXDN.

## Discussion

**Fig. 6:**
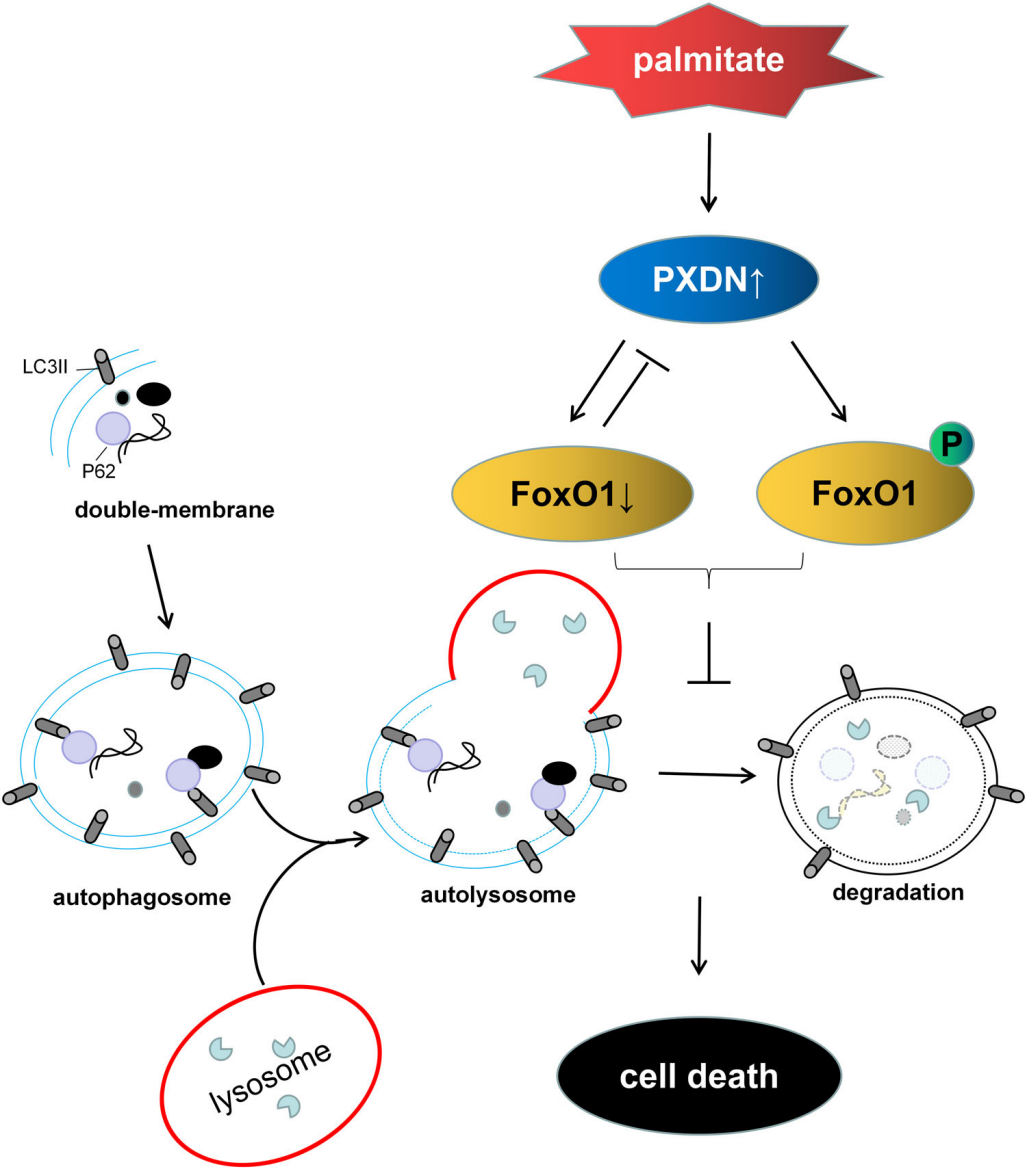
Schematic depiction of the mechanism of PXDN-induced cell death in insulin-resistant cardiomyocytes. The increased protein level of PXDN stimulated by PA impaired autophagic flux to induce cell death in insulin-resistant cardiomyocytes via down-regulating the expression of FoxO1 and promoting FoxO1 phosphorylation.

The main findings of this study were as follows: (1) PXDN increased in the progress of cell death in PA-treated cardiomyocytes; (2) PXDN silence improved autophagic flux and inhibited subsequent cell death induced by PA; (3) The effect of PXDN on autophagic flux was mediated via the expression and phosphorylation of FoxO1; (4) FoxO1 may affect the expression of PXDN (Fig. 6). Our findings firstly demonstrate that PXDN plays a key role in PA-induced cell death via regulating FoxO1 and autophagic flux, while FoxO1 may also affect the expression of PXDN, suggesting that PXDN may play an important role in insulin-resistant cardiomyocytes.

Autophagy is an important cell-protective mechanism in various condition^22,23^, and dysfunction of the autophagic signals is observed in DCM in the previous studies^8,24^. Here in this study, TEM was applied to evaluate the progress of autophagic flux visually, while LC3II (the active isoform of LC3-II, indicating the initiation of autophagy) and p62 (LC3-binding protein, indicating the degradation of autophagosomes) were used as markers to evaluate the degree of autophagic flux. Compared with the control group, increased cell death and impaired autophagic flux were observed in cardiomyocytes stimulated with PA, which was consistent with the work of Bharat et al.^25^.

PXDN is reported to play essential roles in several cardiovascular diseases via catalyzing hydrogen peroxide to form hypochlorous acid and augment oxidative stress^18,26^. Previous studies reported that PXDN significantly increased in the aorta of streptozocin-induced diabetes rats^19^, but the relationship between PXDN and DCM remains unclear. In addition, Increased PXDN under ox-LDL treatment may lead to endothelial cell death via promoting apoptosis by activating p38-mitogen-activated protein kinases/caspase 3 pathway and inducing programmed necrosis by activating β-catenin signaling pathway^20,21^. However, whether PXDN participates in cardiomyocyte death has not been studied yet. In this study, we found PXDN increased in the progress of autophagy flux restrain and cell death induced by PA, and the effect of PA was abolished by PXDN-siRNA transfection, suggesting that PXDN may play an important role in PA-induced cell death.

Transcriptional factor FoxO1 is recently found to involve in the pathology of DCM through regulating autophagy^14,27^. Post-transcriptional regulation is the main regulatory method of FoxO1 activity, and phosphorylation of FoxO1 at specific sites such as Tyr25, Ser256, Ser249, and Ser319 are reported to inactivate FoxO1 by inhibiting the entry of FoxO1 into the nucleus. Ser256, which was detected in this study, is the most widely studied site involving in the inactivation of FoxO1^28^. In addition, it was reported that the decrease of p-FoxO1 (Ser256) was associated with enhanced autophagic signal in insulin-resistant heart^12^. Our study found that FoxO1 expression was decreased by PA stimulation, while si-PXDN reversed this effect and further activated FoxO1 by inhibiting the phosphorylation of FoxO1 at ser256, indicating that PXDN may be able to modulate the expression and activation of FoxO1. Previous studies have shown that phosphorylation of FoxO1 at Ser256 leads to the nuclear exclusion of FoxO1, which triggers its poly-ubiquitination and degradation, among which the phosphorylation of AKT plays an essential role^28^. However, the ratio of p-AKT/AKT was not affected by si-PXDN treatment in our study, indicating that PXDN may affect FoxO1 phosphorylation through another regulatory pathway. In addition, few studies investigated the upstream factors that regulated the expression of total FOXO1 protein level, thus how PXDN affects FoxO1 remains unknown. We believe that genome and proteomic sequencing may be helpful to find possible pathways regulating FoxO1 expression and phosphorylation. Furthermore, the silence of FoxO1 was found to inhibit the expression of PXDN, suggesting that FoxO1 may regulate PXDN in turn to maintain homeostasis. In other words, the decreased expression of PXDN induced by FoxO1 suppression may partially reduce cell damage. Co-IP was conducted to investigate whether FoxO1 could directly bind to PXDN protein. Unfortunately, stable interaction was not found between FoxO1 and PXDN. Since FoxO1, as a transcriptional factor, may regulate the expression of PXDN by binding to the promoter of the gene rather than direct bind to the protein, JASPAR database was applied to predict the potential binding site of FoxO1 and PXDN and results showed that FoxO1 may bind the promoter of PXDN, indicating that FoxO1 may be able to directly target PXDN gene.

Certainly, this study bore some limitations. Firstly, though we have investigated the role of PXDN in vitro, animal models of DCM are demanded to further confirm our results. In addition, the regulatory mechanisms between PXDN and FoxO1 need to be further studied.

In conclusion, this study highlights the pivotal role of PXDN in insulin-resistant cardiomyocytes. Our findings suggest that inhibition of PXDN improves autophagic flux to reduce cell death through suppressing the expression and activity of FoxO1, while FoxO1 may also affect PXDN expression. These findings may develop our understanding of potential mechanisms regarding autophagy in insulin-resistant cardiomyocytes.

## Methods

### Materials

High-glucose Dulbecco’s Modified Eagle’s Medium (DMEM), trypsin-EDTA solution, fetal bovine serum, penicillin, and streptomycin were purchased from Gibco (Thermofisher Scientific, USA). Rabbit anti-PXDN antibody was obtained from Millipore (USA). Sodium palmitate (p9767), rabbit anti-AKT antibody (sab4500797), rabbit anti-p62 antibody (P0067), rabbit anti-LC3 antibody (L8918) were purchased from Sigma-Aldrich (USA). Antibody against phospho-AKT (S473) (ab18206) was purchased from Abcam (UK), while rabbit anti-FoxO1 antibody (CST2880S) and primary rabbit antibody for phospho-FoxO1 (Ser256) (CST9461S) were obtained from Cell Signaling Technology (USA). DC661 (dimeric chloroquine) was purchased from selleck (USA). Glucose assay reagent (F006) was purchase from Nanjing jiancheng bioengineering Institute (Nanjing, China). Reagents for western blot, IgG, and protein A + G Agarose were purchase from Beyotime Institute of Biotechnology (Jiangsu, China). LIVE/DEAD viability kit was obtained from Invitrogen (Thermofisher Scientific, USA). And small interfering RNAs (siRNAs) for PXDN and FoxO1 and negative controls (sequences were listed in Table S1), as well as transfection kit was purchase from RiboBio Co Ltd (Guangzhou, China).

### Cell culture

H9C2, obtained from Cell Bank of China Science Academy (Shanghai, China) was cultured in high-glucose DMEM medium with 10% fetal bovine serum and 0.1% penicillin/streptomycin. AC16 human cell line was obtained from Cellcook Biotech (Guangzhou, China) and cultured in DMEM-F12 medium with 12.5% fetal bovine serum and 0.1% penicillin/streptomycin. The report of AC16 cell line authentication was attached as a supplement material. The incubator was maintained at 37 °C with 5% CO_2_ and 95% air. Subculture was carried out when cells reach 70–80% of confluence. In addition, cells were cultured without serum for 24 h prior to experiments. 1 μM DC661 (dimeric chloroquine) was used to inhibit lysosomal acidification^29^.

### siRNA transfections

siRNA interference was performed as described previously^30,31^. Cardiomyocytes were seeded in six-well plates to reach a 50% confluence and treated in serum-free medium for 24 h with (1) PXDN siRNAs (50 nmol/L each), (2) FoxO1 siRNAs (50 nmol/L each), (3) negative control siRNA (50 nmol/L), (4) both PXDN siRNAs (50 nmol/L each) and FoxO1 siRNAs (50 nmol/L each), respectively. Transfection was achieved using a complement transfection kit according to the manufacturer’s protocol.

### Fatty acid treatment

Fatty acid was applied to induce insulin resistance^32^. 0.028 g of palmitic acid (PA) was dissolved in 2 ml 0.1 N NaOH at a temperature of 75 °C until clear, while 1.8 g of BSA was dissolved in 8 ml 0.9% NaCl at 55 °C. The stock solution of 10 mM PA/18% BSA was prepared by adding the PA solution to the BSA solution, then filtered and stored at −40 °C according to the previous study^33^. 18% BSA solution was prepared as control meanwhile. The stock solutions were heated for 15 min at 55 °C, and then cooled to room temperature before added to the culture medium.

### Glucose consumption

Glucose consumption was measured as previously described^34^. Ten-microliter medium was removed at 0, 12 or 24 h after treatments. Glucose concentration was measured using a glucose assay kit based on the glucose oxidase method. The amount of glucose consumption (GC) was determined by the glucose concentrations of blank wells subtracting the glucose in cell-plated wells.

### Cell viability

LIVE/DEAD viability kit was used to determine cell viability according to the manufacturer’s instructions. Briefly, cells were washed with PBS and then incubated with live green and dead red solutions for 30 minutes at room temperature. Images were acquired using a fluorescence microscope subsequently (Eclipse,Nikon,Japan)^35^.

### Transmission electron microscopy (TEM)

For TEM analyse, cell samples were fixed with 2.5% glutaraldehyde in 0.1 mol/L phosphate buffer overnight at 4 °C, then post fixed in 1% osmium tetroxide and dehydrated in ethanol solutions. HT7700 transmission electron microscope was used to obtain TEM images^36^.

### Western blot analysis

Western blot analysis was performed using standard procedures as described before^37^. Cells were lysed in RIPA buffer spiked with protease and phosphatase inhibitor cocktail. Protein concentration was assayed by BCA Protein Assay Kit before loaded on 8-12% gel. After electrophoresis, the protein was transferred to polyvinylidene fluoride membrane and probed with specific primary and secondary antibodies. The immune complexes were visualized by SuperSignal West Pico Chemiluminescent Substrate and a gel documentation system (Bio-Rad, USA).

### Co-IP

H9C2 cells were lysed in NP-40 buffer and incubated 1 h at 4 °C with IgG and protein A + G Agarose to get rid of non-specific binding. Primary antibody against FoxO1 or IgG was coupled with protein A + G Agarose and incubated overnight at 4 °C. The agarose was collected by centrifugation and washed with PBS for three times. Immunoprecipitated proteins were loaded on 8% SDS gel for western blotting.

### Online database analyses

UCSC (https://genome.ucsc.edu/)^38^ was used to obtain the fasta sequence of PXDN and JASPAR (http://jaspar.genereg.net)^39^ was used to predict the binding sites of FoxO1 and PXDN gene.

### Statistics

Data were presented as means ± SEM. One-way ANOVA with Tukey Kramer test was used when the data were normally distributed, a non-parametric test was used otherwise. SPSS version.23 or Graph Prism 6 were used for multiple statistical comparisons. Differences were considered to be significant at *P* < 0.05.

## Supplementary information

supplement figure legends

Figure S1

Figure S2

Table S1

Table S2

cell line authentication

## Data Availability

All data generated or analyzed during this study are included in this published article [and its supplementary information files].
