# Supplementary material for: PXDN reduces autophagic flux in insulin-resistant cardiomyocytes via modulating FoxO1
Source: Cell Death Dis. 2021 Apr 26;12(5):418. doi: 10.1038/s41419-021-03699-4 (PMC8076187; doi:10.1038/s41419-021-03699-4)
Supplement: Supplementary file 1 — supplement figure legends [file 41419_2021_3699_MOESM1_ESM.doc]

**Figure S1. PXDN silence improves impaired autophagic flux via enhancing FoxO1 in AC16 cells. A.** AC16 cells were treated with 50 μM si-NC or si-PXDN in serum-free medium for 24 hours, and treated with 400μM PA or BSA with or without 1μM DC661 for another 24 hours. PXDN, FoxO1, LC3II and p62 levels were detected by Western blot and quantitative analyses were shown below (n=4). **B.** AC16 cells were treated with 50μM si-NC, si-PXDN, si-FoxO1, or si-PXDN plus si-FoxO1 in serum-free medium for 24 hours, and treated with 400μM PA with or without 1μM DC661 for another 24 hours. PXDN, FoxO1, LC3II and p62 levels were detected by western blot. Quantitative analyses were shown below (n=4). Data were presented as mean ± SEM. One-way ANOVA test was used. *P < 0.05, **P < 0.01, ***P < 0.001

**Figure S2. Stable interaction was not found between FoxO1 and PXDN in H9C2 cells.** H9C2 cells were treated with 400μM PA or BSA for 24 hours. Cell lysates were immunoprecipitated with IgG or anti-FoxO1 antibodies, then analyzed by western blot.
