# Supplementary figures and images for: PXDN reduces autophagic flux in insulin-resistant cardiomyocytes via modulating FoxO1
Source: Cell Death Dis. 2021 Apr 26;12(5):418. doi: 10.1038/s41419-021-03699-4 (PMC8076187; doi:10.1038/s41419-021-03699-4)

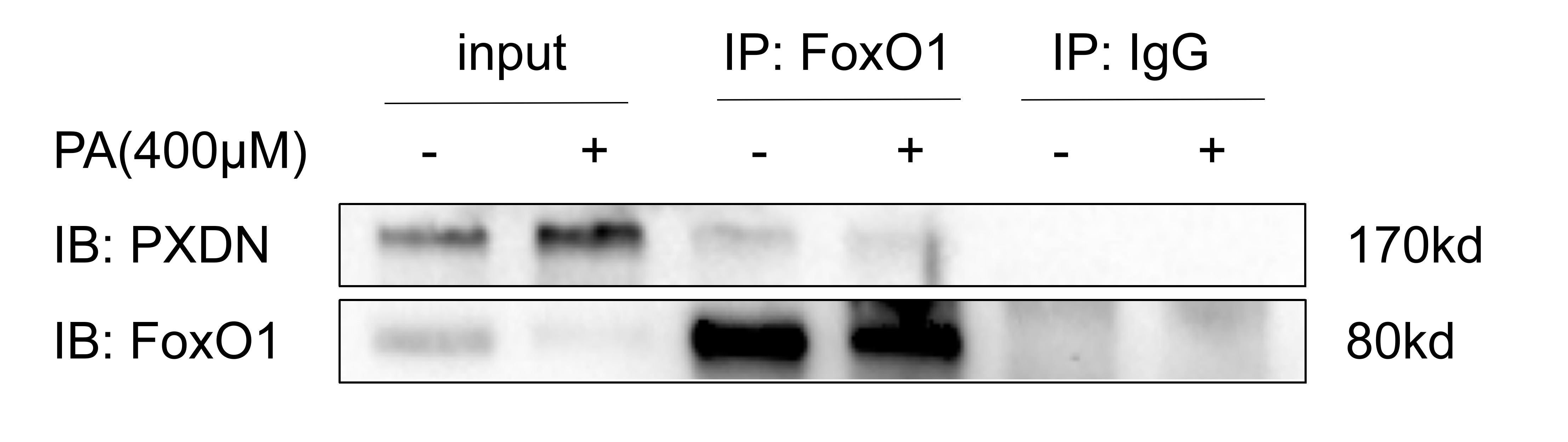

Supplement: Supplementary file 3 — Figure S2 [file 41419_2021_3699_MOESM3_ESM.tif]
