# Supplementary material for: PXDN reduces autophagic flux in insulin-resistant cardiomyocytes via modulating FoxO1
Source: Cell Death Dis. 2021 Apr 26;12(5):418. doi: 10.1038/s41419-021-03699-4 (PMC8076187; doi:10.1038/s41419-021-03699-4)
Supplement: Supplementary file 4 — Table S1 [file 41419_2021_3699_MOESM4_ESM.doc]

**Supplement Table 1: Sequences of small silencing RNAs.**

| Gene name | Species | Sequence(5'-3') | Gene ID |
| --- | --- | --- | --- |
| FoxO1 | rat | CAGCUAUAAAUGCACAUUUA | 84482 |
| FoxO1 | human | CTGCATCCATGGACAACAA | 2308 |
| PXDN | rat | GTGGACTTGAATGGAACAA | 554172 |
| PXDN | human | CCAGTCAATTGACAGGCAA | 7837 |

FoxO1, forkhead box 1; PXDN, peroxidasin
