# Supplementary material for: PXDN reduces autophagic flux in insulin-resistant cardiomyocytes via modulating FoxO1
Source: Cell Death Dis. 2021 Apr 26;12(5):418. doi: 10.1038/s41419-021-03699-4 (PMC8076187; doi:10.1038/s41419-021-03699-4)
Supplement: Supplementary file 5 — Table S2 [file 41419_2021_3699_MOESM5_ESM.doc]

**Supplement Table 2: Predicted binding sites of FoxO1 on PXDN promoter region.**

| Predicted Sequence | Start | End | Strand | Score | Relative score |
| --- | --- | --- | --- | --- | --- |
| TCTTGTTTTTT | 818 | 828 | + | 11.62 | 0.9190 |
| CCATGTTTCCA | 11 | 21 | - | 7.47 | 0.8558 |
| CTGTGTTTCCA | 779 | 789 | - | 6.72 | 0.8443 |
| TTTTGTTGCTA | 1656 | 1666 | - | 5.80 | 0.8303 |
| TCTAGTTTTCC | 1532 | 1542 | - | 5.57 | 0.8268 |
| TGTTGTGTTCT | 1615 | 1625 | - | 5.24 | 0.8217 |
| TTTTTTTTTCA | 828 | 838 | + | 4.55 | 0.8112 |
| TCTTTTTTCCT | 148 | 158 | - | 4.51 | 0.8106 |
| TTTTGGTTTCT | 163 | 173 | + | 4.09 | 0.8042 |
| TGATGTTAACC | 1322 | 1332 | + | 3.97 | 0.8024 |

FoxO1, forkhead box 1; PXDN, peroxidasin
