# Supplementary material for: PXDN reduces autophagic flux in insulin-resistant cardiomyocytes via modulating FoxO1
Source: Cell Death Dis. 2021 Apr 26;12(5):418. doi: 10.1038/s41419-021-03699-4 (PMC8076187; doi:10.1038/s41419-021-03699-4)
Supplement: Supplementary file 6 — cell line authentication [file 41419_2021_3699_MOESM6_ESM.pdf]

# Report of Human Cell Line Authentication

Ⅰ. Sample

Sample Name: labeled as ‘AC16’.

Ⅱ. Method and Procedure

1. PCR is amplified with STR Multi-Amplification Kit (PowerPlex 21D System);
2. PCR products are assayed with 3100 DNA Analyzer (Applied Biosystems®).
3. Amplification of gene COX1 and electrophoresis are employed to survey the species of the sample.

Ⅲ. Results

1. The STR profiles of the cell line sample are in the attached table and figure.
2. The search result in ATCC and DSMZ databases.
3. The electrophoresis figure of gene COX1.

AC16: ①5 loci have tri-alleles . Contamination of other human cell lines are not found (Figure 1 & Table 1). ②Compared the STR data of AC16 cell line in the databases of ATCC and DSMZ, the alleles of AC16 was 90% matched with the alleles of SW900 cells found in ATCC cell bank (Figure 2&3). ③To the data from ExPASy, the sample is AC16. ④The sample is a human cell line. Contamination of other species cells are not found in the sample (Figure 4).

To all above, the sample is a single cell line, and it is AC16 cell line.

*https://web.expasy.org/cellosaurus/CVCL_4U18

Operator: Wanting Jiang Auditor: Xuanyi Liang

Guangzhou Cellcook Biotech Co., Ltd

（Notice:This authentication report is restricted to the cell sold from Guangzhou Cellcook Biotech Co., Ltd, and the date with seal is the date of delivery. ）

Figure 1. STR profiles of AC16 cell line

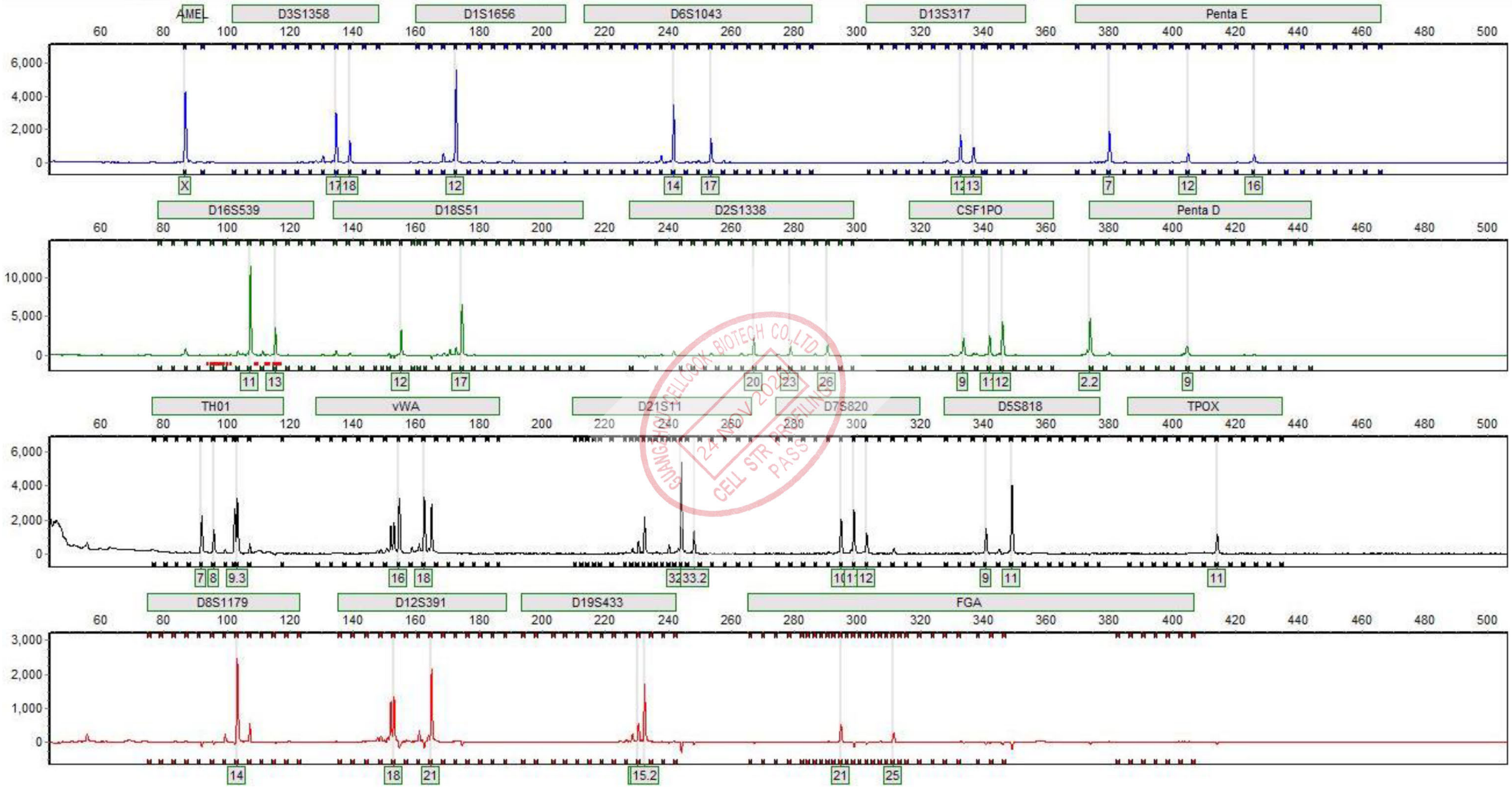

Table 1. STR profiles of AC16 cell line

| 21 | Allele1 | Allele2 | Allele3 |
|---|---|---|---|
| AMEL | X | | |
| D3S1358 | 17 | 18 | |
| D1S1656 | 12 | | |
| D6S1043 | 14 | 17 | |
| D13S317 | 12 | 13 | |
| Penta E | 7 | 12 | 16 |
| D16S539 | 11 | 13 | |
| D18S51 | 12 | 17 | |
| D2S1338 | 20 | 23 | 26 |
| CSF1PO | 9 | 11 | 12 |
| Penta D | 2.2 | 9 | |
| TH01 | 7 | 8 | 9.3 |
| vWA | 16 | 18 | |
| D21S11 | 32.2 | 33.2 | |
| D7S820 | 10 | 11 | 12 |
| D5S818 | 9 | 11 | |
| TPOX | 11 | | |
| D8S1179 | 14 | | |
| D12S391 | 18 | 21 | |
| D19S433 | 15 | 15.2 | |
| FGA | 21 | 25 | |

Figure 2. Search result in ATCC database

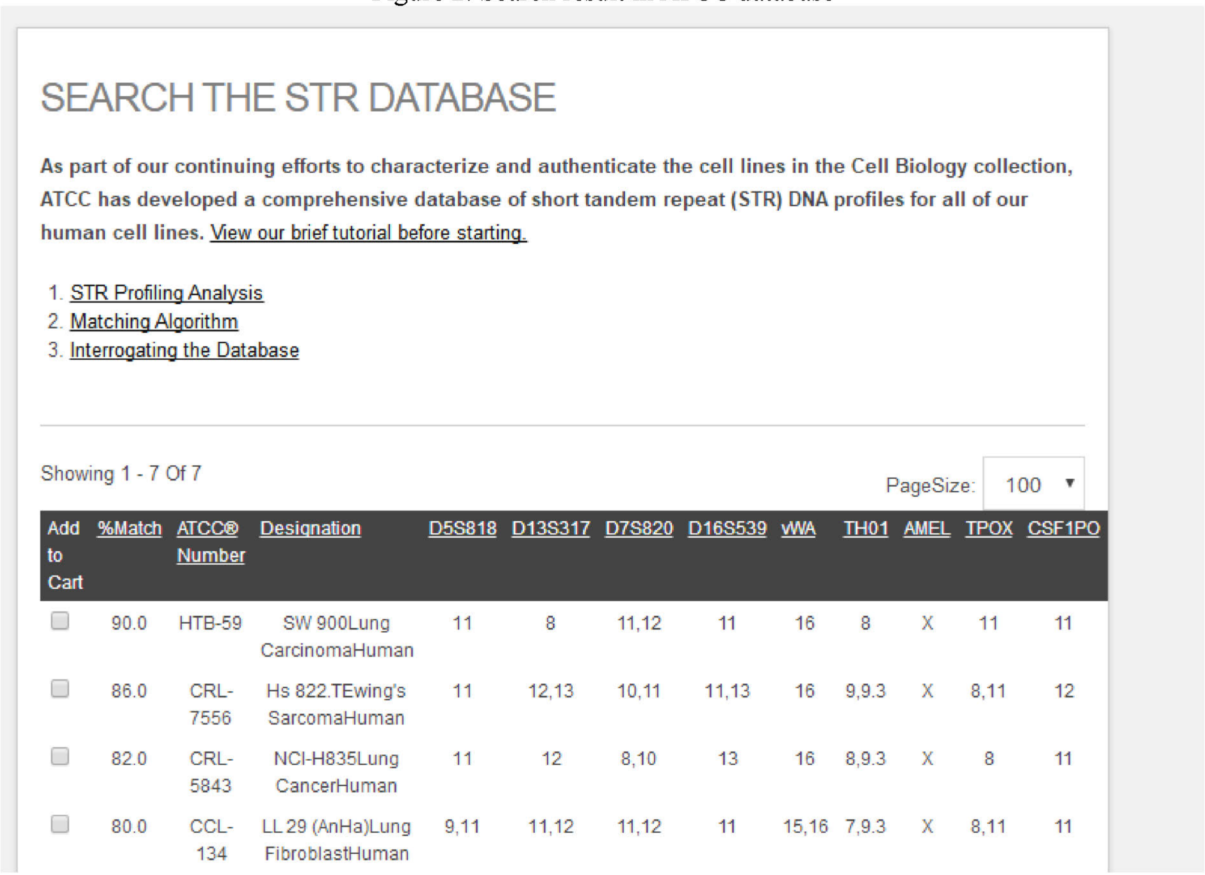

SEARCH THE STR DATABASE

As part of our continuing efforts to characterize and authenticate the cell lines in the Cell Biology collection, ATCC has developed a comprehensive database of short tandem repeat (STR) DNA profiles for all of our human cell lines. View our brief tutorial before starting.

1. STR Profiling Analysis
2. Matching Algorithm
3. Interrogating the Database

Showing 1 - 7 Of 7

PageSize: 100

| Add to Cart | %Match | ATCC® Number | Designation | D5S818 | D13S317 | D7S820 | D16S539 | vWA | TH01 | AMEL | TPOX | CSF1PO |
|---|---|---|---|---|---|---|---|---|---|---|---|---|
| | 90.0 | HTB-59 | SW 900Lung CarcinomaHuman | 11 | 8 | 11,12 | 11 | 16 | 8 | X | 11 | 11 |
| | 86.0 | CRL-7556 | Hs 822.TEwing's SarcomaHuman | 11 | 12,13 | 10,11 | 11,13 | 16 | 9,9.3 | X | 8,11 | 12 |
| | 82.0 | CRL-5843 | NCI-H835Lung CancerHuman | 11 | 12 | 8,10 | 13 | 16 | 8,9.3 | X | 8 | 11 |
| | 80.0 | CCL-134 | LL 29 (AnHa)Lung FibroblastHuman | 9,11 | 11,12 | 11,12 | 11 | 15,16 | 7,9.3 | X | 8,11 | 11 |

Figure 3. Search result in DSMZ database

**Result of STR matching analysis by your data.**

- DSMZ Profile Database -

A graphical presentation is shown at the bottom of this page.

| EV | Cell No. | Cell name | Locus names | | | | | | | | | Figures |
|---|---|---|---|---|---|---|---|---|---|---|---|---|
| | | | D5S818 | D13S317 | D7S820 | D16S539 | VWA | TH01 | AM | TPOX | CSF1PO | |
| | *Query (Your Cell)* | | *9,11* | *12,13* | *10,11,12* | *11,13* | *16,18* | *7,8,9.3* | *X,X* | *11,11* | *9,11,12* | |

Figure 4. Authentication of the species of the sample

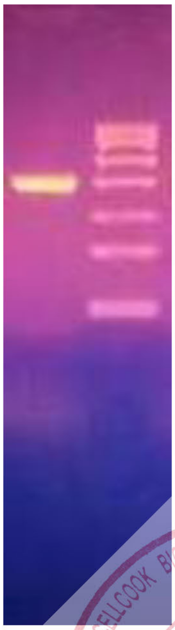

GUANGZHOU CELLCOOK BIOTECH CO.,LTD
24 NOV 2020
CELL STR PROFILING
PASS

M: Marker. As the size of 700, 600, 500, 400, 300, 200 and 100bp from up to down.

Nine species are checked, as follow: *Homo sapiens* 391bp, *Cricetulus griseus*315bp, *Macaca mulatta*287bp, *Cercopithecus aethiops*222bp, *Rattus norvegicus*196bp, *Canis familiaris*172bp, *Mus musculus*150bp, *Bos Taurus*102bp

The sample. The band size is 391bp which matches the size of human.
